# Remotely sensed high resolution irrigated area mapping in India for 2000 to 2015

**DOI:** 10.1038/sdata.2016.118

**Published:** 2016-12-20

**Authors:** Anukesh Krishnankutty Ambika, Brian Wardlow, Vimal Mishra

**Affiliations:** 1 Civil Engineering, Indian Institute of Technology (IIT) Gandhinagar, 382355 Gujarat and ITRA Project: Measurement to Management (M2M): Improved Water Use Efficiency and Agricultural Productivity through Experimental Sensor Network; 2 School of Natural Resources, University of Nebraska, Lincoln, USA

**Keywords:** Environmental chemistry, Hydrology, Geography

## Abstract

India is among the countries that uses a significant fraction of available water for irrigation. Irrigated area in India has increased substantially after the Green revolution and both surface and groundwater have been extensively used. Under warming climate projections, irrigation frequency may increase leading to increased irrigation water demands. Water resources planning and management in agriculture need spatially-explicit irrigated area information for different crops and different crop growing seasons. However, annual, high-resolution irrigated area maps for India for an extended historical record that can be used for water resources planning and management are unavailable. Using 250 m normalized difference vegetation index (NDVI) data from Moderate Resolution Imaging Spectroradiometer (MODIS) and 56 m land use/land cover data, high-resolution irrigated area maps are developed for all the agroecological zones in India for the period of 2000–2015. The irrigated area maps were evaluated using the agricultural statistics data from ground surveys and were compared with the previously developed irrigation maps. High resolution (250 m) irrigated area maps showed satisfactory accuracy (R^2^=0.95) and can be used to understand interannual variability in irrigated area at various spatial scales.

## Background & Summary

Demands for fresh water during the twenty first century will continue to increase to meet the needs of a growing global population. Irrigation water use is projected to increase under climate warming to maintain or increase agricultural production. Irrigation is estimated to use about 70% of world’s total available freshwater for food production using 18% of cultivated area globally^1,2^. Irrigation practices can play a significant role in agriculture production to meet the projected food demand in several parts of the world by maintaining or increasing crop yields under changing climatic conditions. As a result, accurate information of irrigated agricultural water use and its spatial extent and variation is essential for water resource and crop productivity assessments.

In India, agriculture is the largest sector of employment and a significant fraction of total population depends on it for sustenance and economic livelihood through both traditional and modern agricultural practices^3^. However, agricultural production and food security are highly influenced by short-term weather anomalies, episodic extreme events, and long-term changes in air temperature and precipitation^3^. An increase in winter temperature, erratic monsoon season rainfall, extensive use of ground water resources, and absence of effective adaptation strategies are likely to negatively affect crop productivity^4,5^. In the future, the Indian region may experience stress to meet its water demand due to extreme weather and climate events such as droughts and heat waves, specifically in arid and semi-arid regions, where groundwater extraction is prominent for irrigation^3–5^. A remarkable change in agriculture practices occurred in India after 1970, with a massive agricultural expansion called the ‘*green revolution’*^4,6^. This revolution resulted in the agriculture sector benefiting from the introduction and expansion of different types of irrigation, fertilizers, and high yield crop varieties^4,6,7^.

Monitoring irrigation water demands and consumption requires mapping irrigated areas either through agricultural census or using remotely sensing data. The current spatiotemporal extent of irrigated lands and inter-annual change at regional scales in India is still relatively uncertain and available maps are often outdated or prepared with spatially-coarse resolution data. The primary sources of irrigation data in India are the Directorate of Economics and Statistics of the Ministry of Statistics (DES), Ministry of Water Resources (MoWR), and Food and Agricultural Organization of United Nations (FAO). During the past few years, several spatial data sets of irrigated area at global scale have been developed. For instance, the USGS Global Land Cover Map^8^ was developed using 1 km monthly composite of NDVI obtained from Advanced Very High Resolution Radiometer (AVHRR). The Global Map of Irrigation Areas (GMIA) published by the FAO was developed by Siebert, *et al.*^9^ using approximate information of total irrigated area from national information and other data sources (irrigated area per national statistical unit, irrigated area from point, polygon, and raster maps of land cover and other satellite data) at a spatial resolution of 5-arc minutes. Recently, International Water Management Institute (IWMI) released global irrigated area map for a 10-km grid resolution using methods described in Thenkabail, *et al.*^10^. Moreover, Zhao and Siebert^11^ developed crop class based irrigated area maps for India using net sown area and extent of irrigated crops from the census and land use land cover data at 500 m spatial resolution for year 2005. For the Indian region, a high resolution (250–1000 m) irrigation map based on remote sensing data was completed^10,12^ for the Ganga, Indus, and Krishna River basins. Siddiqui, *et al.*^13^ developed irrigated area map for Asia and Africa regions using canonical correlation analysis and time lagged regression, which is available at 250 m resolution for the year 2000 and 2010 and can be obtained from the International Water Management Institute (IWMI, http://waterdata.iwmi.org/applications/irri_area/) portal. However, high resolution (250 m) irrigated area maps that cover the period of 2000–2015 and all the agroecological zones of India are unavailable, which are required for estimation of irrigation water use and hydrologic modelling. Here, we develop annual irrigated area maps at a spatial resolution of 250 m for the period of 2000–2015 using data from the MODIS and high resolution land use/land cover (LULC) information in India.

## Methods

Irrigated area in this study refers to the partial or full application of water at least once to meet minimum annual water requirements for crops in the agricultural area. In India, about 94% of farmers have land holdings smaller or equal to 4 ha (ref. 14). Based on the Cultivable Command Area (CCA), which is an area that is physically irrigated from an irrigation scheme (based on surface or groundwater sources) and is fit for cultivation, irrigation schemes are categorized into major (area more than 10000 ha), and medium (area between 2000 and 10000 ha) projects utilizing surface water sources and minor (area less than 2000 ha)^7^ projects tapping into groundwater resources. Irrigated binary maps are developed in this work where each cell of 250 m resolution represents an approximate area of 6.25 ha and in case of partial or full irrigation, a grid cell was considered 100% irrigated. In India, irrigation expansion has changed remarkably during the last few decades increasing from 20.6 mha in 1950–1951 to nearly 56 mha in 2002–2003 (ref. 7). Due to variations in agro-climatic conditions there is large inequality in the irrigation potential of different states. For instance, south Indian states such as Andhra Pradesh, Karnataka, Kerala, and Tamil Nadu generally have low irrigation potential compared to the other regions, which can attributed to availability of surface and groundwater resources. Additionally, states like Tamil Nadu, Punjab, Rajasthan, Maharashtra, West Bengal and Haryana have already developed over 70% of its major and moderate irrigation potential. Moreover, states like Orissa, Madhya Pradesh, and Gujarat have utilized over 80% of their irrigation potential. States of Haryana, Uttar Pradesh, Bihar, and Tamil Nadu have irrigated area more than 50% of cropped area, while 95% of arable land in the state of Punjab is irrigated. Wheat, sugarcane, and banana are the major irrigated crops in the country with other crops occupying relatively less irrigated area include groundnuts, pulses, cotton, and coarse cereals.

### Normalized difference vegetation index (NDVI) data from MODIS

The Normalized Difference Vegetation Index (NDVI) can be used as an indicator for irrigated area^12,15–17^ as it represents the amount of green biomass with index values varying in response to changes in vegetation conditions^18–20^. MODIS data products are available at different spatial and temporal resolutions providing a daily global coverage of observations with enhanced spatial, spectral, radiometric, and geometric quality for improved mapping and monitoring of vegetation dynamics^21,22^ and can be used to detect unique multi-temporal, spectral vegetation signatures for crop-type mapping^21–24^. The MODIS NDVI composite data are currently available with enhanced vegetation sensitivity with minimal influences from the atmosphere, view and sun angles, clouds, and inherent non-vegetation influences of canopy background and litter. To separate irrigated and non- irrigated areas, MODIS NDVI data with a spatial resolution of 250 m and a 16-day temporal composite period were used to ensure the highest spatial resolution and least amount of cloud contamination. For each year during the study period of 2000–2015, MODIS-NDVI data were projected to the geographic coordinate system and mosaicked using the NASA reprojection tool (mrtweb.cr.usgs.gov). The16-day composite NDVI data were used without any smoothing as smoothing techniques typically result in only subtle increases in overall classification accuracy and some of these techniques lead to large inconsistencies in previous classification efforts^2,25^.

**Figure 1:**
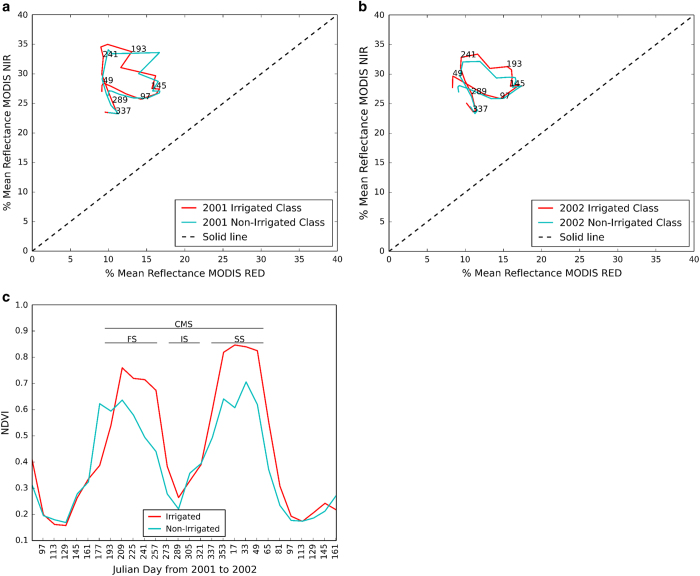
Variation of NDVI in irrigated and non-irrigated areas. (**a**,**b**) Space-time spiral curves (ST-SCs) to study subtle changes in LULC spectral separability for 2001 and 2002 (Julian Day) and (**c**) Temporal profile of NDVI during 2001 and 2002 for irrigated and non-irrigated areas for seasonal pools of complete season(CMS), first season (FS), intermediate season (IS), and second season (SS).

Irrigated and non-irrigated crops show considerable differences in NDVI for the same crop type or cropping pattern. For instance, irrigated crops exhibit higher NDVI especially for corn and wheat^16,17,20,26^, which can be used to separate those from the non-irrigated crops^10,17,21^ (Fig. 1c). Increased soil moisture availability from irrigated water applications during the growing season helps the agricultural crops to attain maximum NDVI exceeding the peak NDVI of non-irrigated crops^16^. The temporal NDVI profile also reflect variations from sowing to harvest and different types of vegetation indices can depict biophysical characteristics of a particular crop type^27,28^. Colombo, *et al.*^29^ showed that Leaf Area Index (LAI) has a positive correlation with NDVI of different crop types, which can be used to identify crops from their phenological phases of vegetation dynamics such as green up, maturity, and senescence^28,30–33^. In addition, NDVI and LAI are strongly correlated, with NDVI saturating (reduced sensitivity) when LAI reaches a value of 4 or more, which is identified as non-crop vegetation. LAI rarely approaches such a value for crop and this would be no issue for irrigation classification therefore NDVI alone can be used to differentiate irrigated from non-irrigated areas as well as crop types. In rainfed areas, NDVI may not achieve saturation during the crop growing period, which also provides a basis to map irrigated areas using NDVI. Moreover, NDVI helps to segregate classes of same crops within an agricultural area, which in turn can be used to differentiate between irrigated and non-irrigated areas due to difference in peak value of NDVI.

### Identifying potential agricultural area and defining cropping schedule

The methodology consists of two main steps as shown in Supplementary Fig. 1: 1) classifying crop types using spectral similarity along the n-dimensional space vectors based on decision rules of physically-interpretable thresholds selected from spectral features, and 2) a decision tree model formulated using the vegetation condition index (VCI)^34,35^ for each pixel in the selected agroecological region to accommodate the weather component in separating irrigated and non-irrigated areas. A LULC map derived from the Indian Remote Sensing (IRS)—P6 AWiFS available at 56 m resolution was used to map agriculture areas under different seasons (Kharif, Rabi and Zaid). Agricultural land located on slopes of 8% or less are suitable for surface irrigation^16^. As a result, areas with a slope greater than 20%, as well as non-agricultural areas were masked from the composite NDVI data. Agroecological zones (http://www.fao.org/docrep/009/a0257e/A0257E02.htm) in India are well defined and mapped using the ArcGIS shape files. The analysis was performed for each zone separately to account for variations in climate, soil types, and phonological cycle of crops. More information on the input data used in our work can be obtained from Table 1.

The crop calendar and growth stages are prepared for each agro-ecological zones using the data from the National Food Security Mission (http://nfsm.gov.in/nfmis/RPT/CalenderReport.aspx). However, natural variations in sowing and harvesting dates may differ because of other factors related to varying topographic conditions, soil moisture in the earlier growth stages, soil salinity intrusion during the dry season, flooding in wet season, general weather conditions, and farmer management decisions. Since vegetation health depends on the region of growth, data preparation and decision tree model development was carried out separately for each agro-ecological zone. A lookup table consisting of average height of crop and major crop types for different agroecological zones was prepared as discussed in Gajbhiye and Mandal^36^. Here average height refers to the difference in crop type value of NDVI achievable during its time of growth in an agroecological zone. Average height data were prepared based on visual analysis of different crops profiles of peak NDVI value for rain fed and irrigated time period from published literature. Since locations of specific crop type were not available NDVI height were prepared from published literature. Thus each threshold value was assigned for each crop type based on the NDVI in hierarchal manner. The scheme of implementation at first was based on three broad grouping of crops in the Kharif, Rabi, and Zaid seasons. Identification of each crop (Rice, Wheat, Cotton, Maize, Sorghum, Pulses, Barley, Soybean, Sugarcane, Pearl Millet, Jowar, Groundnut, Sesamum, Bean, Gram and Berseem) was done on the basis of crop-specific average height at the time of sowing, seedling growth, tillering, flowering, ripening and harvest.

Multi-temporal NDVI profiles were extracted from the time-series MODIS data of each selected area for each 250 m pixel over agricultural land, while n-dimensional temporal NDVI data were used to separate probable crop type using a lookup table. Emergence and senescence periods for a crop were identified using cumulative NDVI departure (departure was estimated against the mean value and when cumulative departure is minimum the date was identified) for a water year (i.e., from month of June of current year to the month of May next year (June to May)). A NDVI composite (NDVIC) was created for every water year by layer stacking MODIS images. The NDVIC selected samples were used to define crop type based on their NDVI temporal profiles. For each NDVIC sample, the start of growing season, duration and height information were used to assign the crop type based on the condition prepared in the corresponding lookup table. The obtained crop type class information was then used as a training sample to segregate crops of spectral similarity, which was finally used in the irrigated/non-irrigated classification algorithm. Since each pixel may represent more than one crop type because most of the crop fields are small in comparison with the pixel size of the data, a spatial sample size of 250 m which is equivalent to a pixel of MODIS NDVI was selected uniformly throughout the entire agro-ecological zone. Considering the boundary of each district, the selected pixels were spaced at an average distance of 4 km. Maximum attainable NDVI value range considered for cropped area is normally in range between 0.1 and 0.8, and corresponding height was scaled in a range between 1 and 100. The created crop classes were used as an end member signature for further crop type classification of the entire agroecological-zone. Classification of NDVIC (Normalized Difference Vegetation Index Composite) was performed using Spectral Correlation Mapper (SCM) where each end member defined as a crop is considered as a spectrum representative of the spectral class property of n-dimension space vector. The SCM is a modification of the Spectral Angle Mapper (SAM) approach^37^ with the major difference from latter technique being that the data are standardized and centered on two spectra, i.e., the reference and the image spectrum, which allows a better spectral difference by using pairs of deviation and by detecting false positive. The basic limitation of the SAM is its inability to distinguish negative and positive correlations because only absolute values are considered. The SCM is a derivative of the Pearson correlation coefficient that excludes negative correlation and varies between −1 and 1^38^. Hence SCM presents a more accentuated contrast between targets of interest (i.e., irrigated and non-irrigated crops) and highlights pixels representing negative correlations allowing the algorithm to distinguish the targets of interest more accurately.

### Decision tree irrigation model

Shahriar Pervez, *et al.*^16,20^ and Ozdogan, *et al.*^15^ demonstrated that the NDVI threshold approach was promising for identifying irrigated areas. A decision tree classifier was developed by applying a NDVI threshold for individual class clusters (crop type belong to same class obtained from the SCM) (Supplementary Fig. 2). The framework was based on the fact that individual ‘seasonal pools’ (refers to growing stages of a specific crop) of crop type (i.e., spectral subclasses of individual crop and rotating crop) were assessed and used to establish a NDVI threshold to differentiate irrigated from non-irrigated areas. This approach addresses the temporal dynamics of cropping frequency, which has an unique advantage in that it does not require calibration to match the reported irrigated area from agricultural census data using a technique such as that of Shahriar Pervez, *et al.*^16^. Moreover, the NDVI threshold was obtained by transforming the original NDVI into the Vegetation Condition Index (VCI; Kogan^34^). VCI is generally used for drought monitoring which needs separation of short-term weather related fluctuations from long-term ecosystem changes in vegetation. Since VCI can provide information on stressed or unstressed condition of a crop, it can be used to separate irrigated and unirrigated areas. Normalized VCI can be used to identify NDVI differences between irrigated and non-irrigated areas. To date, the VCI has mainly been used to monitor weather impacts on vegetation such as drought detection and monitoring^34,35,39^. VCI of every individual crop class was analyzed for each year to get the mean VCI (MVCI). Here, the VCI is considered a key proxy for separating an irrigated and a rainfed crop from the same crop system because it includes weather-related information expressed by NDVI fluctuations in the intra- and inter-annual NDVI time-series data.

A NDVI threshold (*NDVI*_irclass_) value was identified for each crop type. Initially, maximum value of *NDVI*_iclass_ i.e., *NDVI*_imclass_ was segregated based on each crop class obtained from SCM for the every seasonal pools. The mean NDVI was obtained for each class (*NDVI*_iclass_) using maximum of *NDVI*_iclass_ from every seasonal pool of the crop growing period. Here irrigated area separation is based on crop type of the same class or cropping pattern. Moreover, it is evident that the stress condition can be identified using the maximum vegetation index value of a particular crop class compared to the index value of the same crop class in a water-stressed condition. MVCI was estimated using equation ii and multiplied by 100 to measure percentage change of the current *NDVI*_mp_ from the *NDVI*_iclass_. The percentage variation was then used as a proxy for the stressed condition for the same class types. This stressed percentage was then used as a multiplier coefficient to separate irrigated from non-irrigated area, which was given in equation (1). Therefore, *NDVI*_irclass_ was best suited to separate cropped area under irrigation.

(1)NDVIirclass=(MVCI×NDVImp)+NDVImp

where

(2)MVCI=(NDVImp−NDVImclassNDVImclass)andNDVImclass=1n∑i=1nNDVIimclass

where *NDVI*_irclass_ is a threshold for separating irrigated class *NDVI*_iclass_ value present in each class of SCM, *NDVI*_imclass_ mazimum value of *NDVI*_iclass_, *NDVI*_mp_ is the minimum value of *NDVI*_imclass_, *MVCI* is the Mean Vegetation Condition Index, and *NDVI*_mclass_ is the mean NDVI value for particular class. Each seasonal pool and each crop type was evaluated separately and using a minimum *NDVI*_irclass_ value equivalent or greater than 0.2 of NDVI for irrigated area. The irrigated area uses *NDVI*_irclass_ as threshold when a minimum difference of 0.15 or greater exists between values of *NDVI*_iclass_, and NDVI_mp_. The details of decision tree classification are presented in Supplementary Fig. 2.

For post classification analysis, Space Time Spiral Curves (ST-SCs) were used to verify irrigated area for spatial and temporal consistency which further differentiate irrigated and non-irrigated crops even both falls in same region as explained below (Fig. 1c). ST-SCs use two-dimensional feature space (i.e., NIR and RED reflectance) of the MODIS data to track near continuous changes in class spectral behaviour over time and space. As presented in Thenkabail, *et al.*^10^ ST-SCs curve demarcate the class territory as for each class that is rain-fed in ‘brightness territory’, the water class in ‘wetness territory’ and the irrigated class in ‘greenness territory’. Nonetheless, it can categorize changes in magnitude difference of features like built-up, forest, and crop growth. The ST-SCs can identify when two classes have similar spectra and provide excellent class separability on most dates from different classes and even show differences in irrigated and non-irrigated crop class for one or more dates (Fig. 1a,b). In addition to this the time series trend in irrigation with respect to rainfall was also analyzed for agro-ecological zones and states.

## Data Records

The study area extends spatially from 68° E to 97° E and 6° S to 37° N, which covers the entire Indian region. Irrigation data sets are available to the public through an unrestricted repository in a Geotiff format (Data Citation 1). The data is in binary format i.e., 0 and 1. The value 1 represents irrigated area with a pixel size of 250 m. The data provide a static representation of irrigation during a water year and cover the entire period of 2000–2015. The data repository delivers only irrigation raw data obtained for the entire Indian region. The repository updates of irrigated area will be done every year upon the availability of new data set.

## Technical Validation

**Figure 2:**
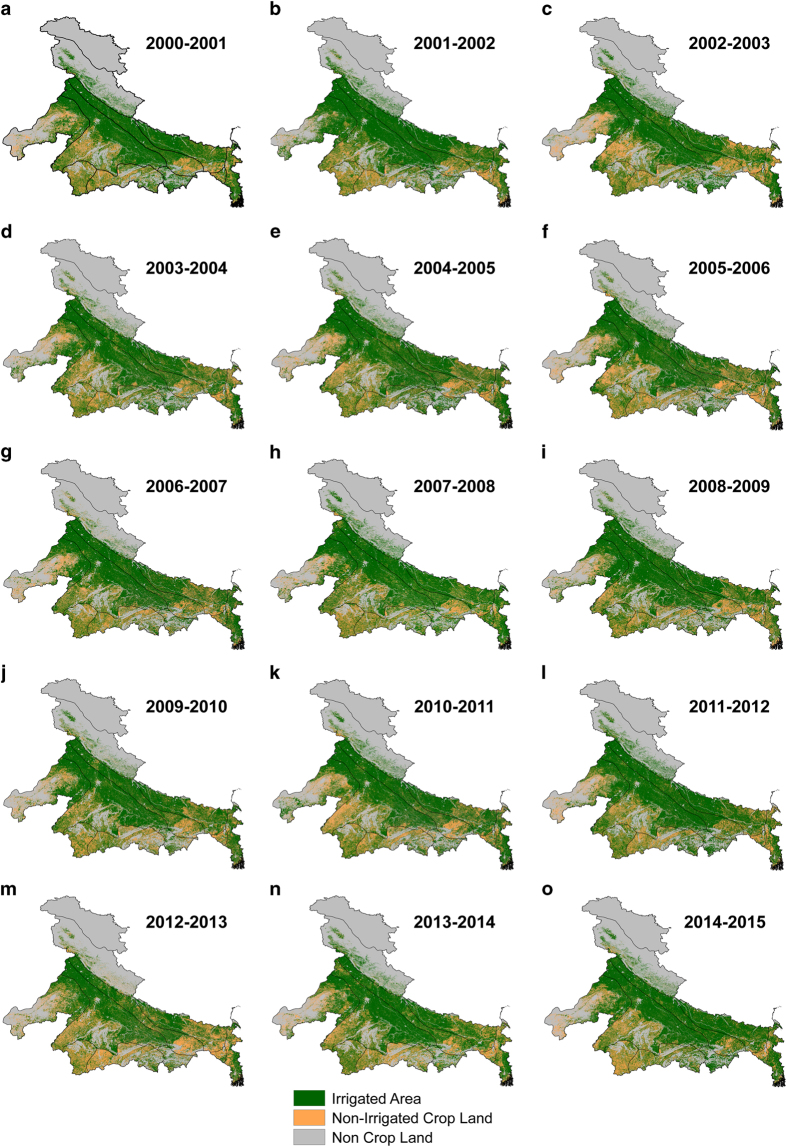
Changes and variability in irrigated area in the Indo-Gangetic Plain. Temporal variation of irrigated area in the Indo-Gangetic Plain for water years for the period of 2000–2015.

**Figure 3:**
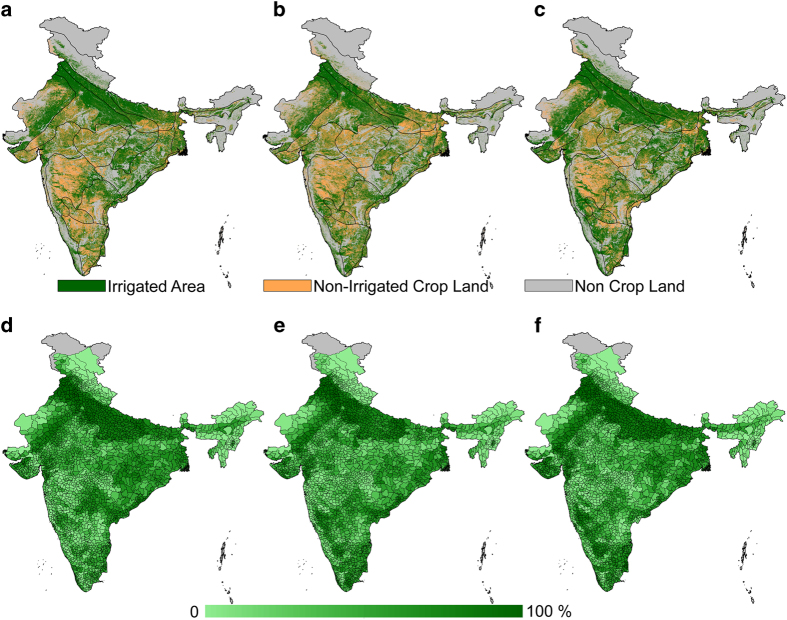
Irrigated area in India during the selected drought years. (**a**–**c**) Irrigated (green) and non-irrigated (brown) areas in India for 2000, 2012, and 2015, and (**d**–**f**) taluk based irrigated area (%) estimated by aggregating the 250 m irrigated area based on MODIS NDVI for 2000, 2012 and 2015.

The application of the model over the 15-year study period resulted in 15 annual irrigation maps for the time period of 2000 to 2015 (Figs 2 and 3). The validation section presents two sets of results: 1) temporal validation of irrigated area with agricultural statistics data for the entire country and for each state (DES) and 2) an irrigated area comparison of the Ganga River Basin. Since no ground based observed irrigated area data are available, we provide accuracy assessment against the DES data for each state, which is based on ground based survey for irrigated area. Moreover, we selected the Ganga basin for the analysis as it is one of the most irrigated basins in the world. Both surface and groundwater based irrigation is being used in the Ganga basin, which can provide us an excellent case for the evaluation of our approach. The irrigated area for the Ganga basin during 2001–2002 was developed by Thenkabail *et al.*^10^ using a series of 500 m MODIS images. The Ganga Basin irrigated area comparison was done using space time spiral curves (SC-STs) introduced by Thenkabail, *et al.*^10^ to check near continuous changes in class behaviour over time and space to match the irrigation area obtained for the years 2001 and 2002. The accuracy of irrigated area obtained from 500 m MODIS reported by Thenkabail, *et al.*^10^ is based on fuzzy approach and the produced six irrigated-area classes had an accuracy of 100, 75, 84, 56, 79 and 100 (%) with a mean accuracy of 82%. The accuracy assessment of these maps was challenging due to lack of ground truth and site-specific information on irrigated areas, however, better information on accuracy can be achieved through direct comparison with DES data based on ground survey of irrigated areas. These comparisons allow us to examine the accuracy in terms of temporal correlation and spatial variability of irrigated class at the 250-m resolution. Since the irrigated area maps were developed for a water year, the evaluation represents the same period.

### Irrigated area comparison for the Ganga River basin using SC—STs curve

The irrigated area for the Ganga River basin was reported to have an area of 26.9 mha during the period of 2001–2002 based on the 500-m resolution data developed by Thankbeil *et al.*^10^. Our estimate for the same period for the Ganga River basin for irrigated area was 30.75 mha. Some differences in the two estimates can be attributed to classification errors and difference in spatial resolutions (250 and 500 m) between the two classified maps. Moreover, uncertainties in irrigated area estimation in 500 and 250 m maps may also be due to insufficient ground reference points that are needed to avoid misclassificiation. Spatial resolution may be an important factor as a 500 or 250 m pixel is considered fully irrigated if there is partial irrigation in small fields within a pixel. We adopted the space- time spiral curves method for validating irrigated area, were ST-SCs have the capability to demarcate spectral class territory and the validation was carried out for ‘greenness territory’, which contains the irrigated and non-irrigated areas. Irrigated and non-irrigated areas were well separated during different time period of growth for the years 2001 and 2002 (Fig. 1a,b). Figure 1 shows a good agreement between our estimates and results in other reports that show a similar trend, where ST-SCs during 2001 and 2002 of irrigated area significantly overlap one another for most of dates. Given that 2002 was a drought year, the greenness of agricultural land was reduced because of less water availability in non-irrigated areas compared to 2001, which was evident in the results shown in the Fig. 1. For instance, a noticeable difference can be seen in both images on 241th day, which may be due to the fact that 2002 was a drought year with low greenness as discussed in Thenkabail, *et al.*^10^.

### Multiyear irrigated area

A multi-year irrigated area for different states was estimated using statistics data of Net Irrigated Area (NIA) obtained from DES (http://www.aps.dac.gov.in/LUS/Index.htm) for the period of 2000–2013. A detailed description of data used as input for validation are given in Table 1. To our knowledge, the DES dataset is the most reliable and provides areal information on the temporal variation of irrigated area to validate our result. Moreover this dataset is used by several government agencies for adoption and mitigation efforts in response to weather fluctuations and extremes^6,40^. The temporal variation in irrigated area in the DES data and MODIS 250 m irrigated area maps for all India is shown in Table 2. Our results showed an overestimation in irrigated area estimated using 250 m MODIS NDVI data (Table 2). Since our estimates are based on 250 m spatial resolution, irrigated area may be overestimated due to small land holdings in India. This highlights the need of higher resolution irrigated area maps in India that can resolve the boundaries of majority of small land holdings.

The effect of classification algorithm, and threshold considered here for irrigation mapping are somewhat subjective and may have uncertainty associated with them, hence it is important to communicate to users this information before using these irrigated agricultural land data sets. The misclassification may introduce positional uncertainties even in the cropping area, which could result in selecting the incorrect NDVI threshold for defining irrigation. Another source of potential error could be attributed to the 250-m spatial resolution of the MODIS source data, where non-irrigated areas smaller than the pixel size are often classified as irrigated and can only be distinguished with higher spatial resolution data. However, the multi-year irrigation data set produced in this study was robust enough to capture the overall decline of irrigation during drought years (Fig. 3), which can be attributed to less shown area during drought years. The results validated with published literature^10^ using a different technique give substantiate evidence in support of the representative of the accuracy of the overall irrigation patterns obtained using our methods (Tables 2 and 3).

Since ground based survey data of irrigated area are available at state level, we compared estimated mean irrigated area between 2000 and 2013 from the 250 m MODIS data against the DES for each state (Table 3). Using the 250 m MODIS NDVI, the irrigated area for all the states showed root mean square error (RMSE) of 0.83 mha and coefficient of determination (R^2^) of 0.95. These results indicate the effectiveness of our approach to map irrigated area against ground based survey of DES. We notice that the states of Andhra Pradesh, Assam, Jharkhand, Orissa, Rajasthan and Uttar Pradesh showed an overestimation in estimated irrigated area that ranged between 1.02 and 2.31 mha. The bias in our estimates is random as in some states irrigated area was overestimated while in other it was underestimated. However, in most of the states, our results provided better estimates of irrigated area in comparison to the product developed by the International Water Management Institute^13^ (IWMI) [R^2^=0.76 and RMSE=7.99 mha] at the same resolution (Table 3). Apart from the spatial resolution (250 m), which may not be able to resolve boundaries of small land holdings^41,42^, bias in estimated irrigated area may also be caused due to variation in peak NDVI. Since NDVI is an indicator of vegetation health, the effectiveness of our approach may be higher in semi-arid or arid regions, where water is a limiting factor for crop growth. For instance, in humid and sub-humid regions, the difference in peak NDVI, that is used as a threshold to identify irrigated and non-irrigated areas may not be high leading to errors and bias in the classification. Since a majority of agroecological zones of India fall in water limited conditions, we assume that our method is effective for India. Other than these factors (resolution and difference in peak NDVI), management related factors (differences in soil productivity, fertilizer application, and sowing time) can also influence the classification of irrigated and non-irrigated regions in India. Notwithstanding sources of uncertainty, our results provide better estimates of irrigated area in comparison to the other existing dataset (IWMI).

### Standardized anomalies in rainfall and irrigated area

A comparison of the first difference time series of rainfall and irrigation is presented to show the trend and response of irrigation to rainfall variations. The areas were grouped by agroecological region, state, and country and the time series difference data were evaluated at a spatial scale of 250 m. Supplementary Fig. 3 presents the temporal irrigation pattern and first difference map of rainfall with irrigation for the Indian region. The latter were normalized by subtracting the mean from the actual value and dividing it by corresponding standard deviation using annual average of rainfall and irrigation area for a particular region. Declines in irrigated area were fairly constant for some years with values ranging between −1.46 and −2.60 mha, however, other years 2003–2004, 2005–2006 and 2012–2013 showed a larger decrease in irrigated area with values ranging from −6.21 to −13.38 mha. One reason for the latter case is that the preceding year had a pronounced rainfall deficit as illustrated in Supplementary Fig. 3c. In addition, Panda and Wahr^43^ reported during 2012 year that the GRACE-derived negative water storage anomaly for the post monsoon and monsoon were less compared to previous years. In addition to that post monsoon of 2012 (October to December) and pre monsoon (January to May) of 2013 having a consistent temporal behaviour of groundwater level anomaly was noticed by in situ data for the years 2011 and 2012. Terrestrial and ground water storages withdrawal were also less during 2012 drought year (may be because of less sown area), which could be a possible reason to decrease irrigation for this time period.

A notable finding in the Indo-Gangetic plain is shown in Fig. 2 for irrigation during the 2002 water year where irrigation was not affected by the observed rainfall deficit, which was calculated as a positive difference of 2.15 mha from other obtained techniques as discussed in Thenkabail, *et al.*^10^. Other studies also showed the same trend for the same water year^10^, which can be attributed to substantial groundwater-based irrigation in the region. In contrast, for the Indo-Gangetic plain, the spatial extent of irrigated area was reduced during the 2012 drought year because of a decline in net cropped area due to rainfall deficit in the early monsoon season (June) [Figs 2 and 3]^43^. Interestingly for the state of Punjab, a negative anomaly in irrigated area was seen for most of the years since 2008 (Supplementary Fig. 3b), and had almost recovered to a comparable pre-2008 irrigated area by 2015. Reports claim this state had faced consecutive dry years^43^, which may have attributed to such trend.

A more detailed view of irrigation at different time period is shown in Fig. 3, which shows irrigation condition during water surplus year and water deficit years of 2012 and 2015 to represent recent periods. A clear difference in irrigated area estimated using 250 m MODIS NDVI can be noted from Fig. 3a,b, in which most of northern and eastern plain have higher variability in irrigated area, whereas in southern belt and all over the country there was an increase in irrigation during 2015 year compared to 2000 (Fig. 3). A Taluk-based irrigation map (political boundary of India, which divides a district into smaller regions) was prepared to show the applicability of our data for agencies interested in seeing how irrigation was affected at a regional scale. Figure 3d–f well represent severe drought year of 2012, which was the lowest irrigated area during the period of 2000–2015. The entire eastern and lower northern plain was affected by severe drought. For the three targeted time periods, temporal variations due to rainfall anomalies were reflected in our irrigation maps.

**Figure 4:**
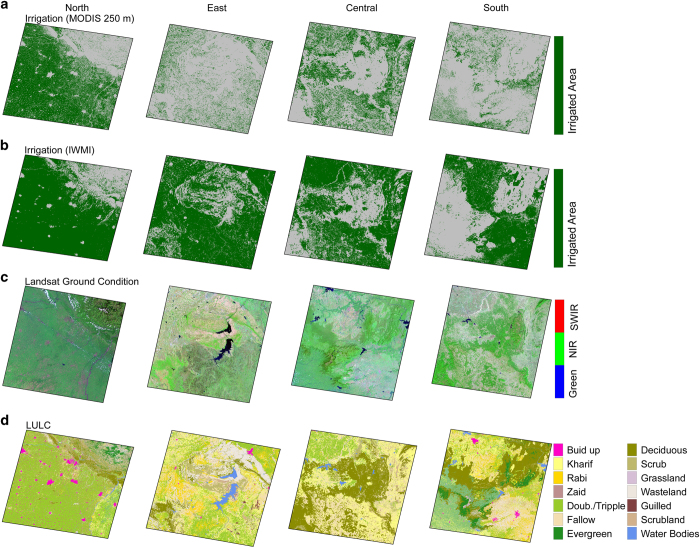
Comparison of irrigated area developed using 250 m MODIS NDVI and data from IWMI at the same spatial resolution for specific land use land cover classes. (**a**) Irrigated area developed using the 250 m MODIS NDVI, (**b**) irrigated area from 250 m resolution map from IWMI, (**c**) Regional views depicted by Landsat ETM data, and (**d**) AWiFS Land Use Land Cover (LULC). Each column from left to right represent north, east, central and southern region of India.

More detailed regional representation of irrigation mapping results for four locations with different climatic and regional LULC patterns across India are shown in Fig. 4. We considered Landsat (Table 1) satellite data collected during the 2000–2001 crop growing season to show surface conditions (at higher spatial resolution) of the obtained irrigated area and LULC for more specific thematic image information. The 250-m irrigated area estimates from the MODIS NDVI and IWMI data (250 m) were used to compare irrigated area in the selected locations in the north, south, east and western parts of India with respect to different LULC types to check whether any of these irrigated area data (250 m MODIS and IWMI) were subjected to under or over estimation (Fig. 4). A noticeable difference in the MODIS-based 250 m data and that obtained from IWMI was found suggesting an overestimation of irrigated area in the IWMI data, which is consistent with the comparison against the ground based DES data (Fig. 4). A substantial decline in irrigation was shown in MODIS 250 m results compared to the IWMI data. Disparities in both the datasets can be primarily attributed to classification approaches^13^. We also considered central and south regions where the majority of area under either forest and agriculture, and IWMI based irrigated area maps overestimated the irrigated areas in several of these forested locations that were none, whereas the 250 m MODIS-derived maps were free from such effect due to removal of such area prior to the irrigation classification phase. Availability of ground reference data of irrigated area can be used for accuracy assessment of irrigated area maps developed at higher resolution. These findings highlight the importance of moderate resolution 250 m irrigated area maps for a country like India that has small irrigated land holdings. The irrigated area maps developed using the moderate resolution satellite information and the classification methodology outlined in this paper can be used to estimate irrigation water demands and water resources planning. It is also important to emphasize that irrigated area estimation should be conducted annually to get better understanding of influence of climatic variations on irrigation.

## Usage Notes

A simple but effective classification method for mapping irrigated areas was presented in this paper that uses seasonal peaks of *NDVI*_iclass_ in a hierarchal decision model for mapping irrigated area for India annually between 2000 and 2015. The classification method was calibrated separately for each agroecological region considering the region’s ecological potential and short-term weather fluctuations. The SCM classification for crop mapping along with the application of decision tree model produced relatively accurate irrigated maps that were comparable with irrigated area reported by other studies for Indian region during the same period and also well depicted its inter-annual variability during water deficit years. Due to the lack of traditional ground reference data of known irrigated fields, areal statistical data were used to verify the classified irrigated area between 2000 and 2013 and SC-ST curves for cross verifying the published result for the 2001–2002-time period. Irrigation area results from the maps produced in this study were consistent with the irrigated area of the DES and other published reports, which provides confidence that our technique is robust enough to be applied in other parts of the south Asian region. Uncertainty and bias in the estimated irrigated area can be further reduced by using a high resolution NDVI data that can capture the variability in the small land holdings in India.

In general, high resolution irrigation data are required to study irrigation water requirement, for applications such as cropping pattern changes, combining hydrologic modeling to determine ground water recharge in arid and semi-arid regions, assessment of water demands at catchment scale, characterizing spatial and temporal variation of crop yields caused by irrigation, temporal and spatial pattern of evapotranspiration in highly irrigated area. For example, high resolution maps can be used to assess the fraction of areas under irrigation in Taluks in India as displayed in Fig. 3. Figure 3 highlights the substantial irrigation fluctuations in areas of north and southern regions. Around 19% decline in irrigated area was noticed during 2012 drought year as compared to the previous years, which shows that the region was affected by this severe drought event. A sub-region scale irrigation climatology can also be developed using this annual times-series irrigation data set. The dataset can also be used to estimate irrigation water demands and water management under the water stress conditions. The irrigated area maps can be used for planning in water resources and agriculture in India.

Irrigated area maps are presented in tiff format for each water year (2000–2001, 2001–2002 and 2014–2015). The maps can be easily imported in ArcGIS for analysis of irrigated area in any region in India.

## Additional Information

**How to cite this article:** Ambika, A. K. *et al.* Remotely sensed high resolution irrigated area mapping in India for 2000 to 2015. *Sci. Data* 3:160118 doi: 10.1038/sdata.2016.118 (2016).

**Publisher’s note:** Springer Nature remains neutral with regard to jurisdictional claims in published maps and institutional affiliations.

## Supplementary Material



Supplementary Information

## Figures and Tables

**Table 1:** Data sets used for irrigated area mapping as input and validation.

**Data**	**Source**	**Spatio-temporal resolution**	**Remarks**
MODIS-NDVI	mrtweb.cr.usgs.gov	250 m, 16 day composite	To Develop Crop Mask, VCI threshold for irrigation
Crop Calendar	nfsm.gov.in/nfmis/RPT/CalenderReport.aspx	Federal state wise, Seasonal(Kharif, Rabi and Zaid)	To segregate different crop based on season
Agroecological Zones	www.fao.org/docrep/009/a0257e/A0257E02.htm	1:5172413 Scale, 20 Zones	To Separate regions with different climate, soil type and crop rotation
Land Use Land Cover -IRS—P6 AWiFS	bhuvan.nrsc.gov.in/bhuvan_links.php	56 Meter	To remove classes other than Crop
SRTM—DEM	earthexplorer.usgs.gov/	90 Meter	To exclude areas with greater than 20%
DES—Irrigation	eands.dacnet.nic.in/	Federal state and country wise irrigated area data for the period of 2000–2012	For validation with obtained irrigation Data
IWMI—Irrigation data	waterdata.iwmi.org/applications/irri_area/	250 m, 2000 and 2010	For comparison with obtained irrigation Data
Landsat −7 ETM +	earthexplorer.usgs.gov/	30 Meter	To show ground condition of growing season

**Table 2:** Comparison of all India irrigated area (mha) from of statistical data (DES) and developed data at 250 m spatial resolution using the MODIS NDVI in this study.

**SL. no**	**Water year**	**DES net irrigated area (mha)**	**MODIS 250 m irrigated area (mha)**
1	2000-01	55.31	77.56
1	2001-02	56.93	76.16
2	2002-03	53.89	82.14
3	2003-04	57.06	73.96
4	2004-05	59.22	74.71
5	2005-06	60.69	65.51
6	2006-07	62.60	76.52
7	2007-08	63.18	73.92
8	2008-09	63.63	73.18
9	2009-10	61.93	71.50
10	2010-11	63.59	75.19
11	2011-12	65.26	78.71
12	2012-13	66.10	64.75

**Table 3:** State wise mean irrigated area from DES, MODIS NDVI (250), and IWMI (250) for the period of 2000–2010.

**SL. no**	**Federal state**	**DES net irrigated area (mha)**	**MODIS**	**IWMI**
			**250 m irrigated area (mha)**	**250 m irrigated area (mha)**
1	Andhra Pradesh	4.40	6.08	19.60
2	Arunachal Pradesh	0.05	0.12	0.11
3	Assam	0.15	1.17	1.65
4	Bihar	3.29	2.71	10.71
5	Chandigarh	0.001	0.001	0.002
6	Chhattisgarh	1.25	3.34	10.05
7	Dadra & Nagar Haveli	0.006	0.005	0.037
8	Delhi	0.02	0.02	0.07
9	Goa	0.03	0.05	0.14
10	Gujarat	3.79	4.42	12.06
11	Haryana	2.98	3.09	5.56
12	Himachal Pradesh	0.11	0.28	0.72
13	Jammu & Kashmir	0.31	0.36	2.12
14	Jharkhand	0.13	1.40	6.70
15	Karnataka	2.99	3.95	13.55
16	Kerala	0.39	0.58	0.59
17	Madhya Pradesh	6.19	5.60	27.00
18	Maharashtra	3.25	3.59	23.26
19	Manipur	0.05	0.06	0.12
20	Meghalaya	0.06	0.12	0.24
21	Mizoram	0.01	0.07	0.005
22	Nagaland	0.07	0.12	0.01
23	Orissa	1.66	3.37	10.65
24	Pondicherry	0.02	0.02	0.04
25	Punjab	4.06	4.38	6.44
26	Rajasthan	6.03	8.34	20.6
27	Sikkim	0.01	0.03	0.01
28	Tamil Nadu	2.75	3.27	10.34
29	Tripura	0.06	0.13	0.29
30	Uttaranchal	0.34	0.48	1.00
31	Uttar Pradesh	13.19	14.28	28.13
32	West Bengal	3.08	2.57	8.28
Total		60.75	74.14	220.22
