# Supplementary material for: Remotely sensed high resolution irrigated area mapping in India for 2000 to 2015
Source: Sci Data. 2016 Dec 20;3:160118. doi: 10.1038/sdata.2016.118 (PMC5170598; doi:10.1038/sdata.2016.118)
Supplement: Supplementary Information [file sdata2016118-s2.doc]

**Supplemental Information:**

**Remotely sensed high resolution (250m) irrigated area mapping in India (2000-2015)**

**Anukesh, KA^1^, Brian Wardlow^2^, Vimal Mishra^1*^**

1. Civil Engineering, Indian Institute of Technology (IIT) Gandhinagar and ITRA Project: Measurement to Management (M2M): Improved Water Use Efficiency and Agricultural Productivity through Experimental Sensor Network.
2. School of Natural Resources, University of Nebraska, Lincoln, USA

- Corresponding author: vmishra@iitgn.ac.in

**Supplementary Figures**
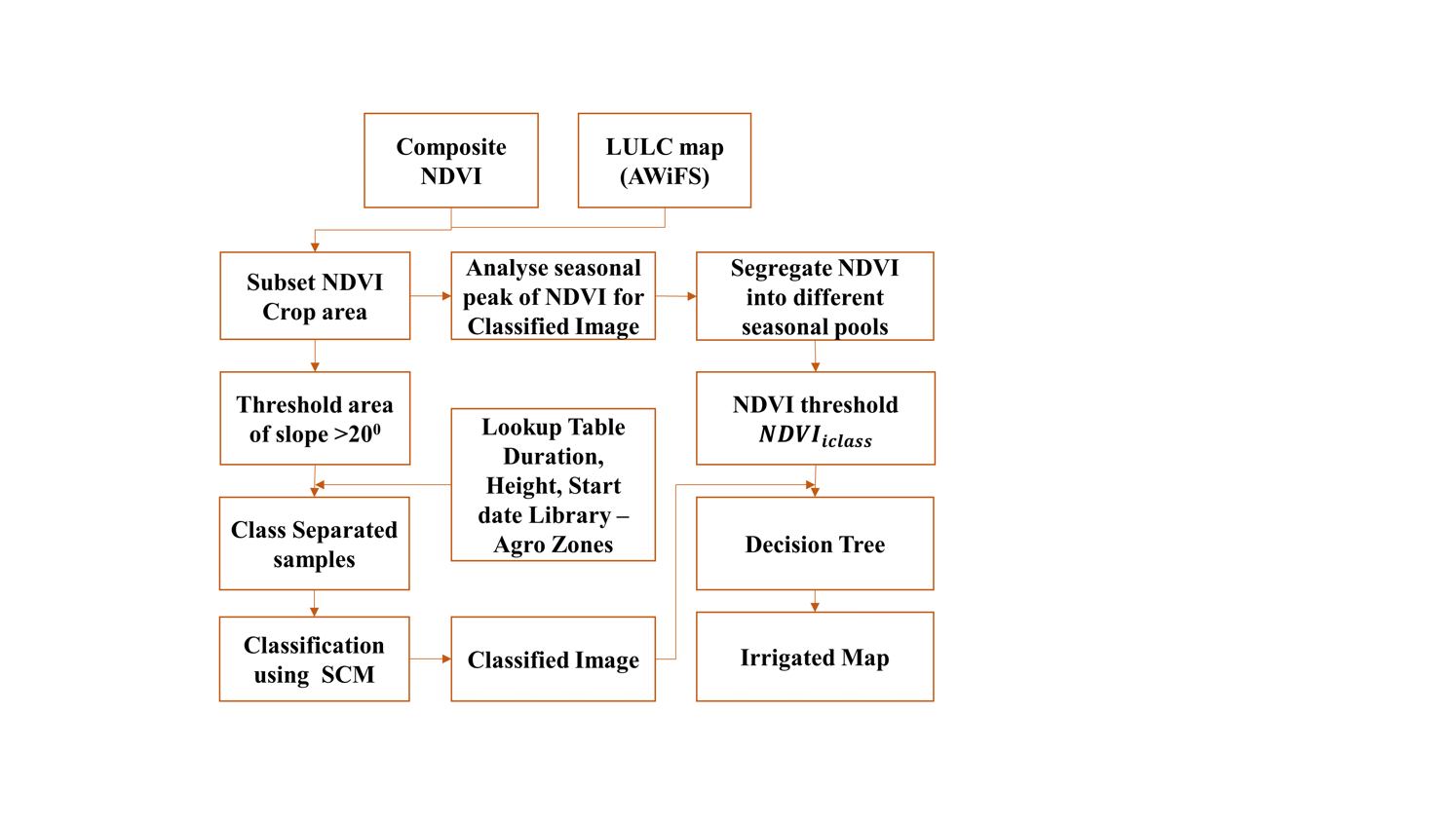


Figure S1. Flow chart of the major steps in the proposed irrigation mapping algorithm.


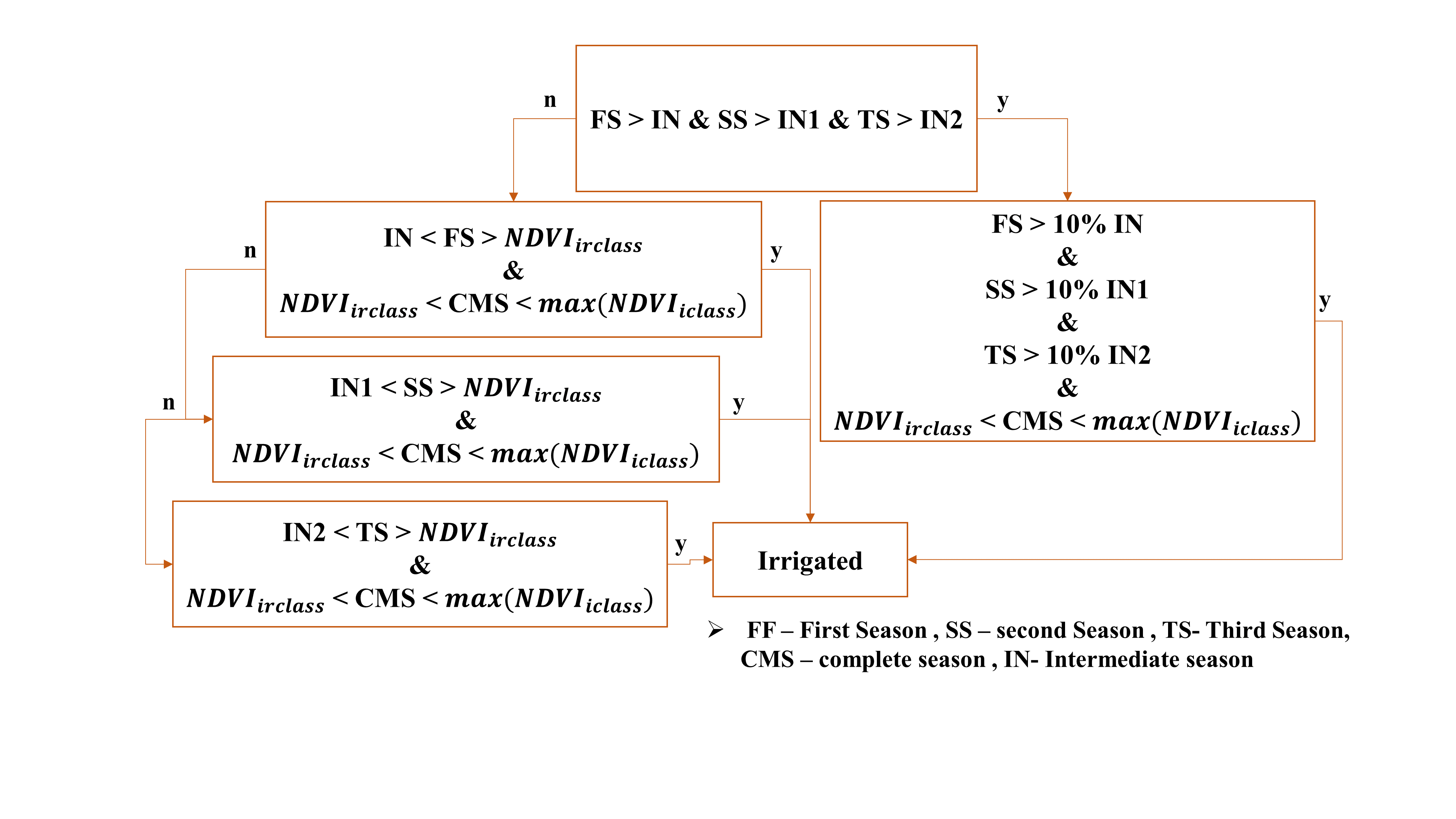


Figure S2. A simplified decision tree for a single class to get a binary map of irrigation. Here each IN is calculated for the respective crop senescence period.


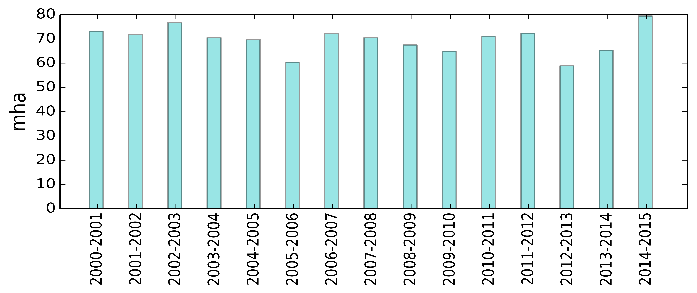

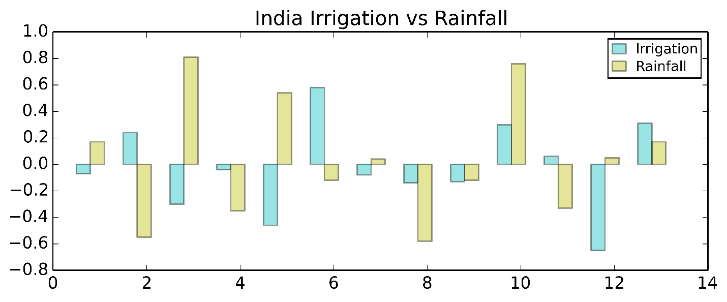


**A**

**B**

**c**


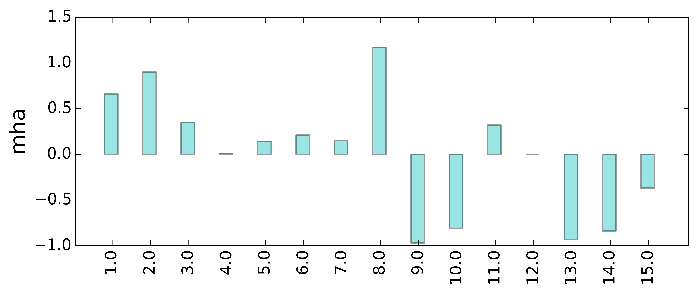


Figure S3. a) Irrigation variation of India in million hectare area between 2000 and 2015, b) first difference of standardized anomaly of all India irrigated area and rainfall and c) temporal variation of irrigated area anomalies based on water year between 2000 and 2015


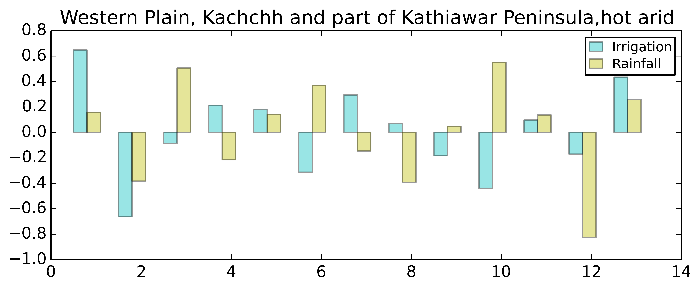

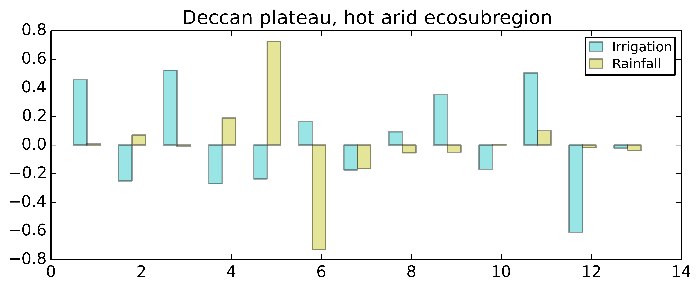


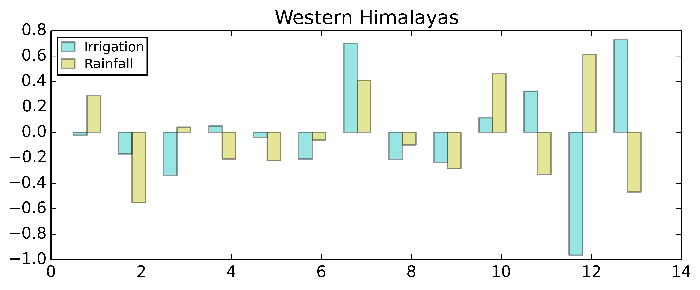

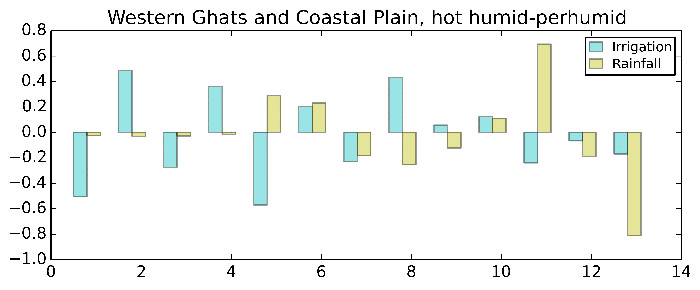

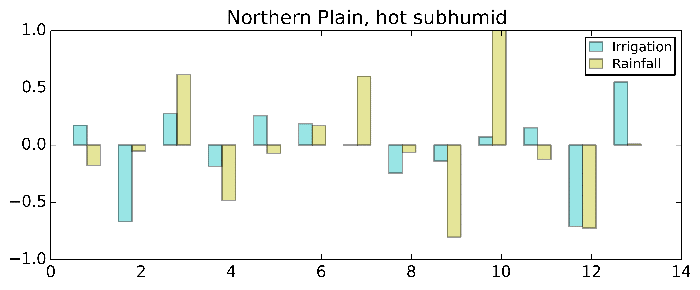

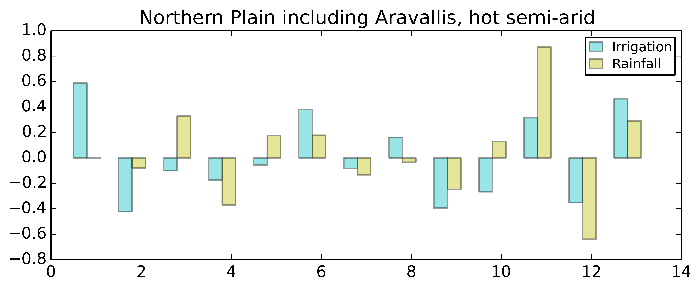

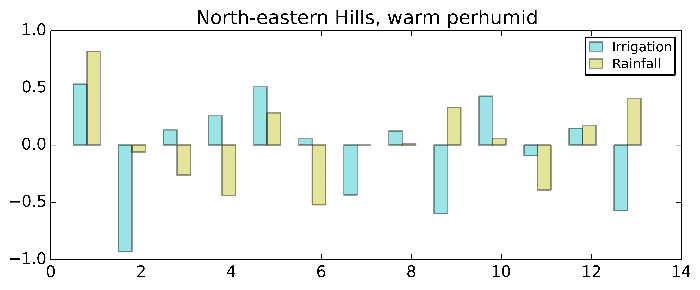

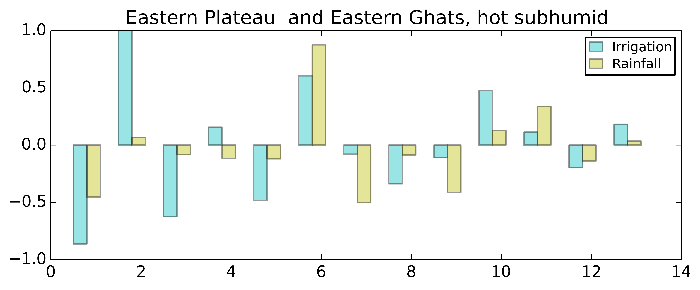

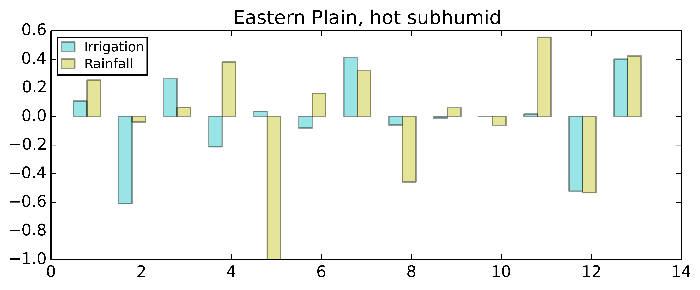

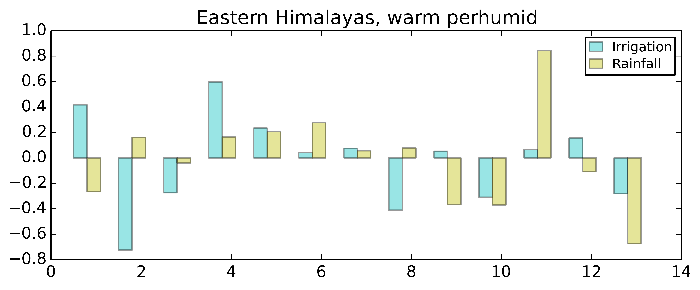

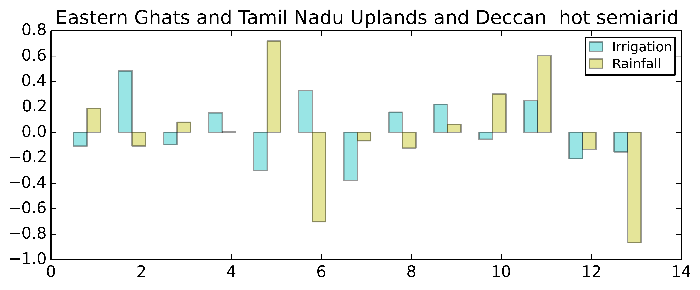

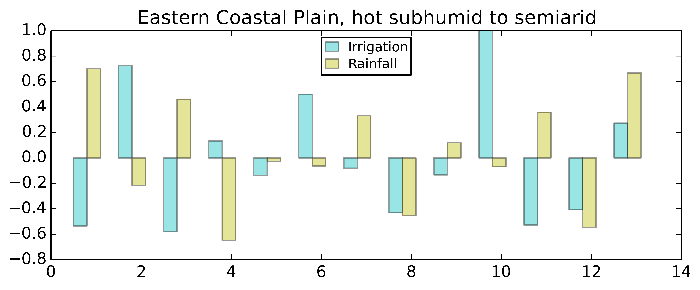

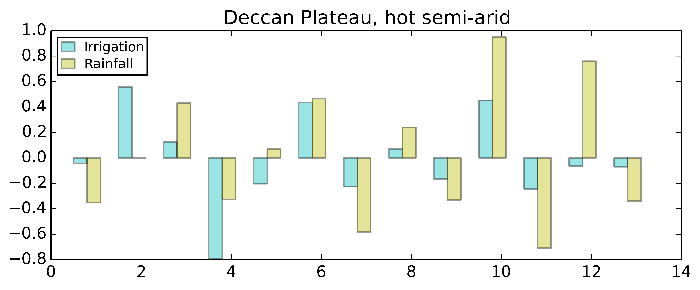

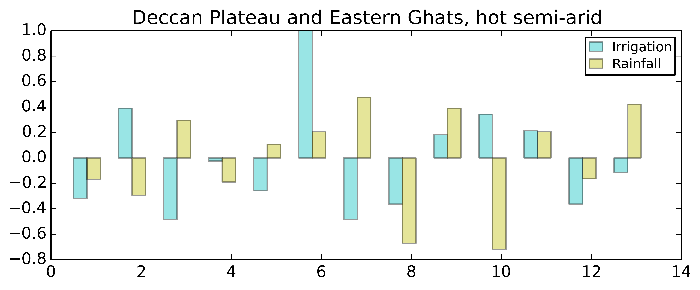

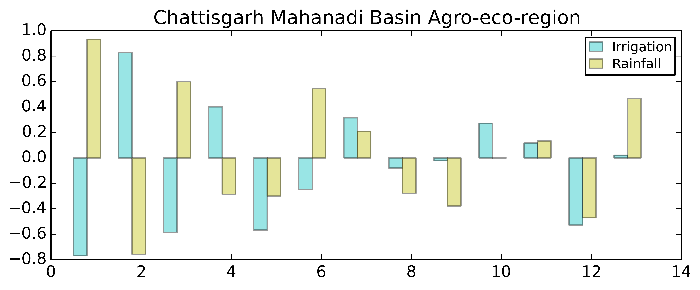

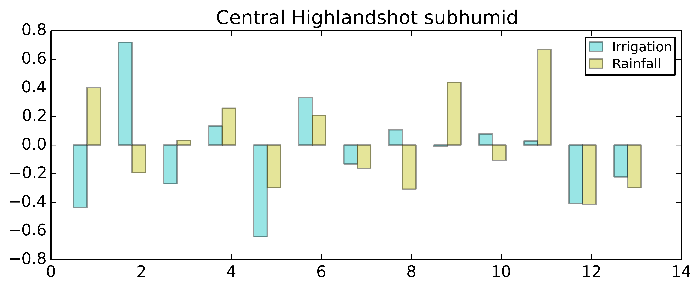

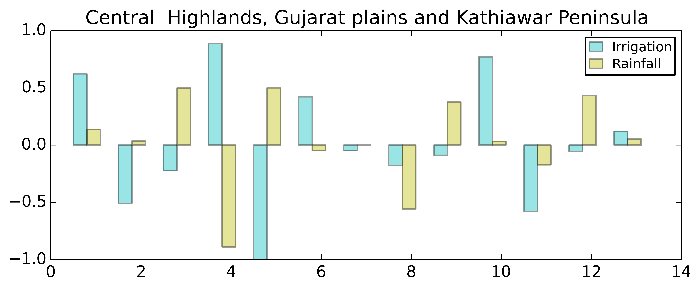

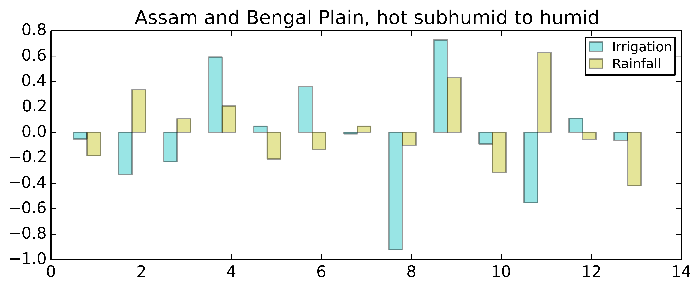


Figure S4. First difference of standardized anomaly of irrigated area and rainfall for the agroecological regions in India


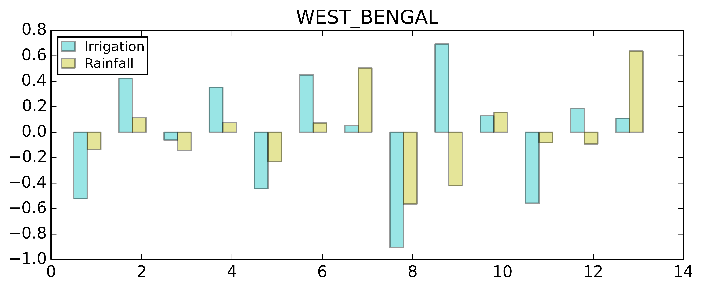

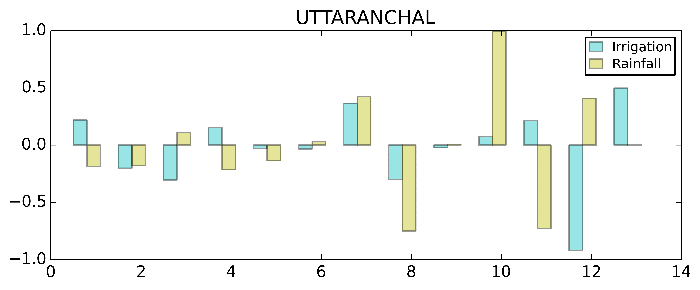

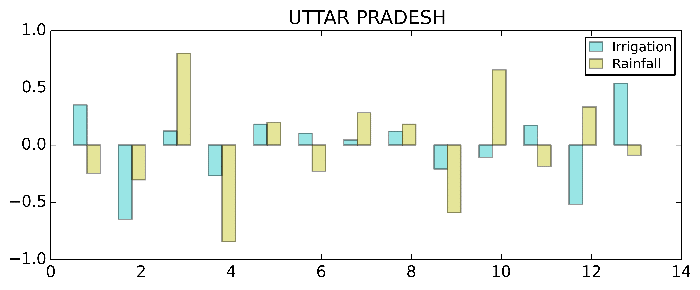

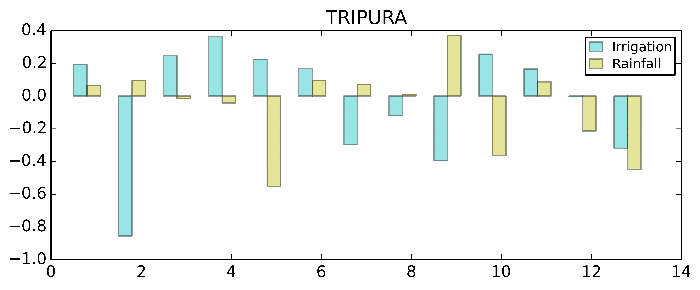

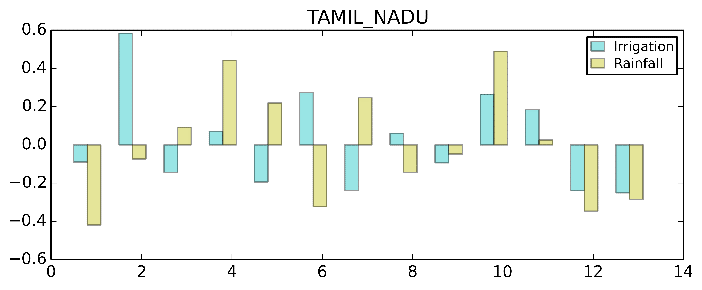

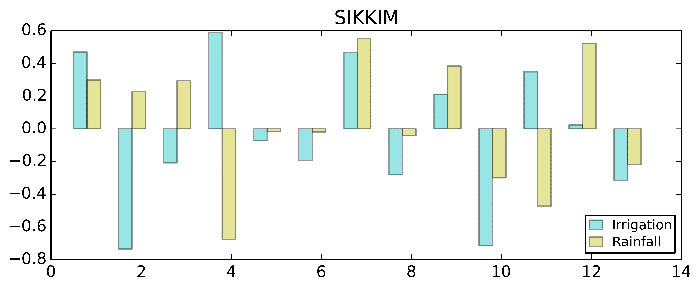

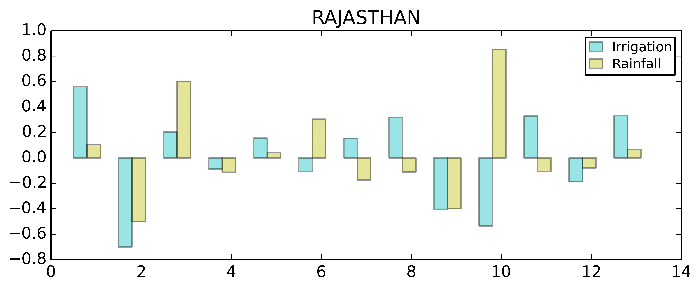

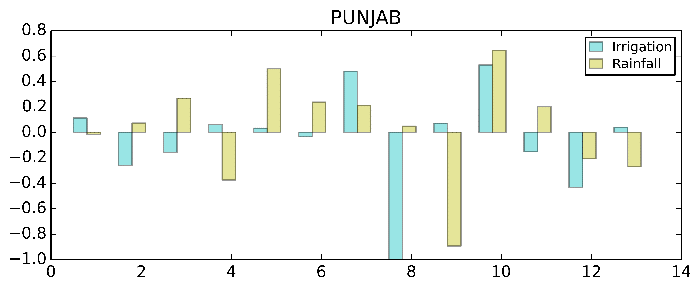

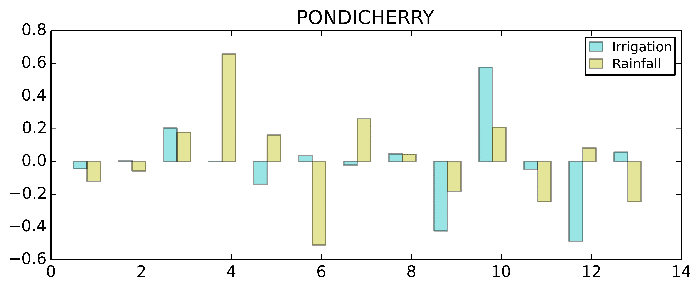

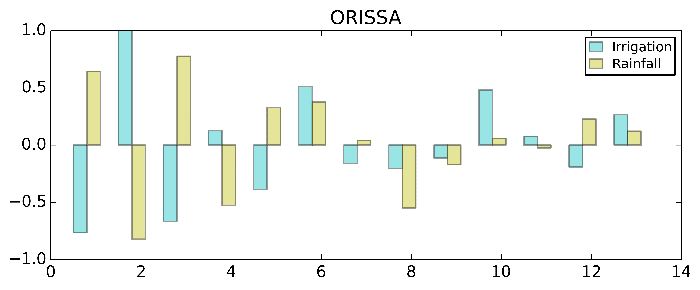

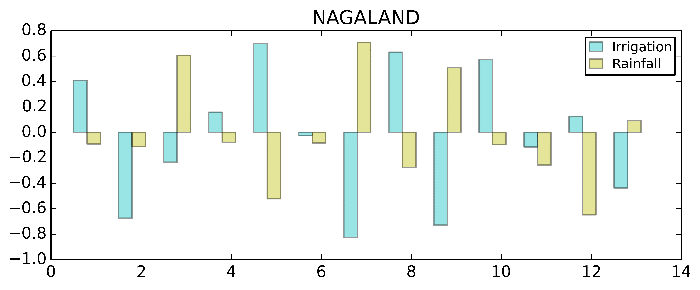

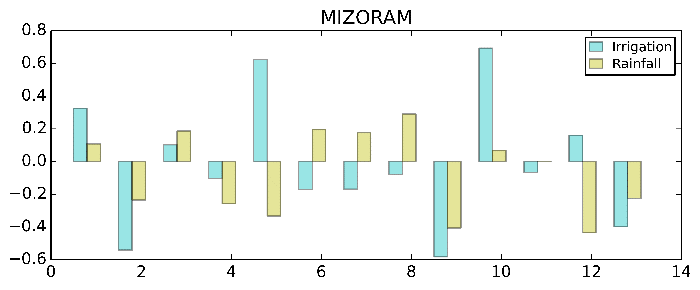

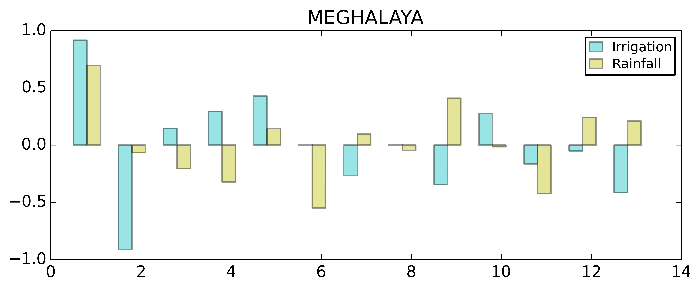

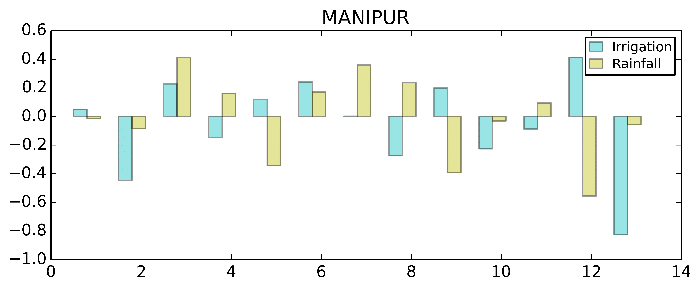

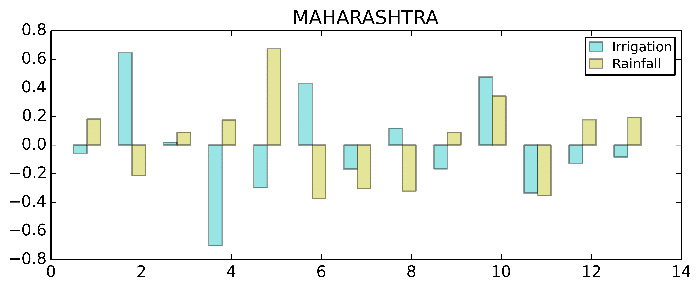

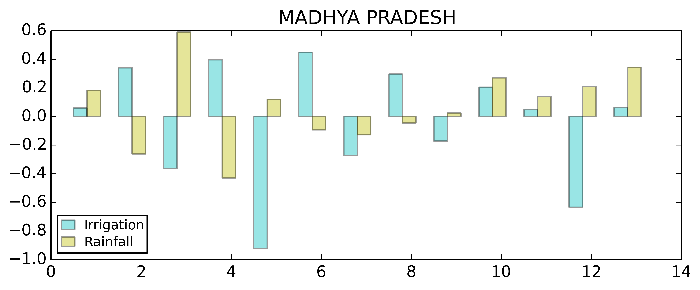

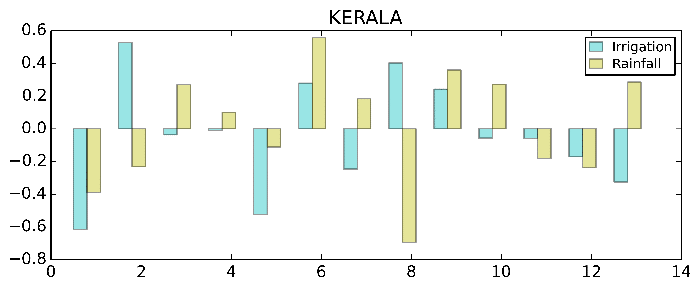

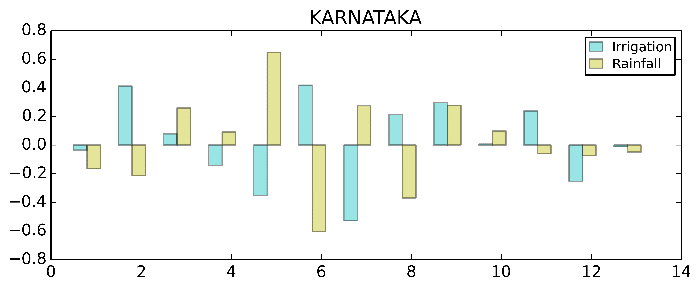

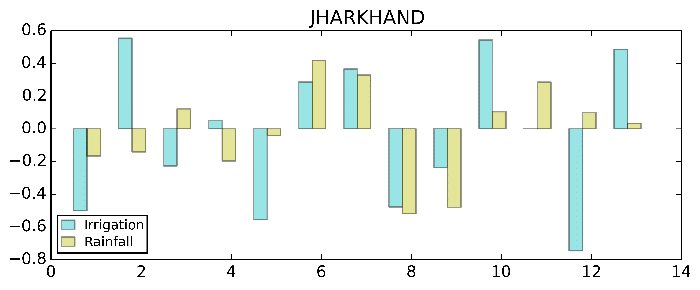

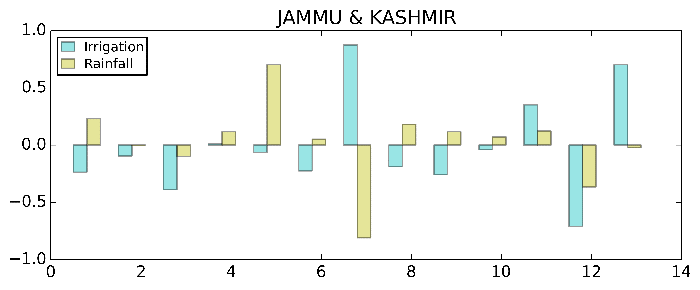

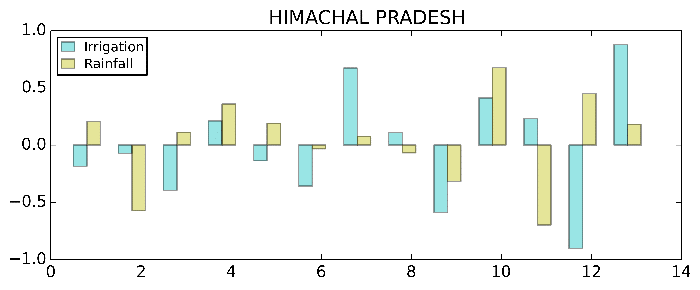

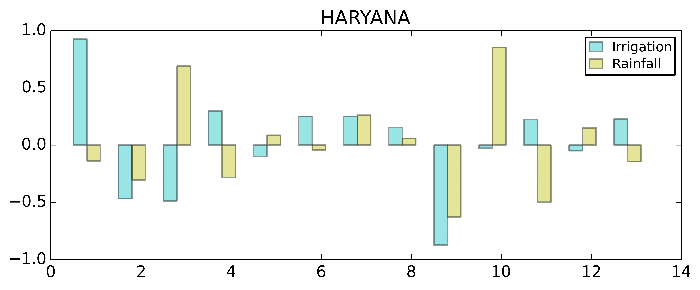

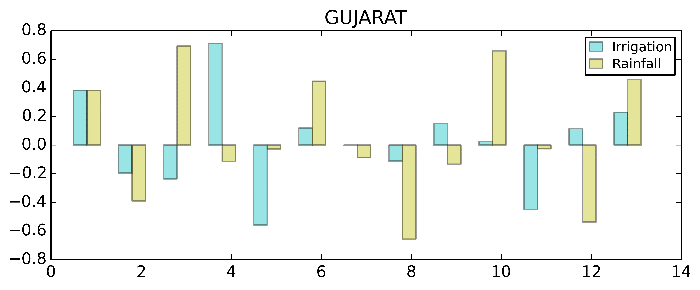

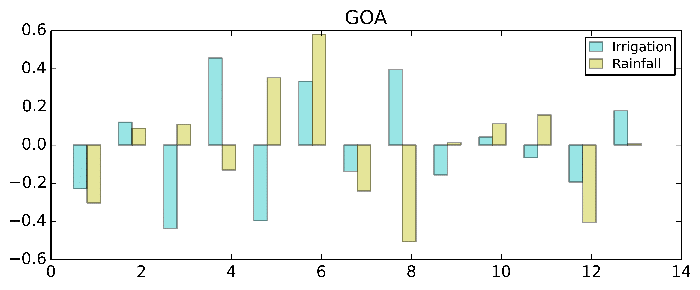

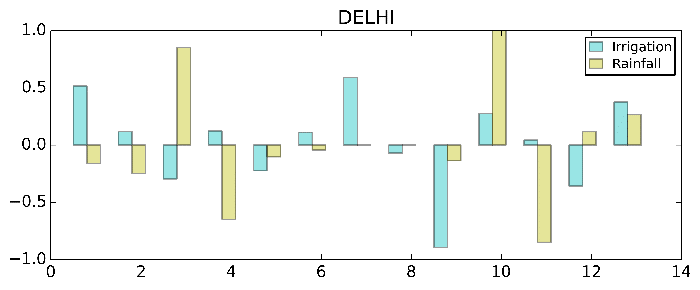

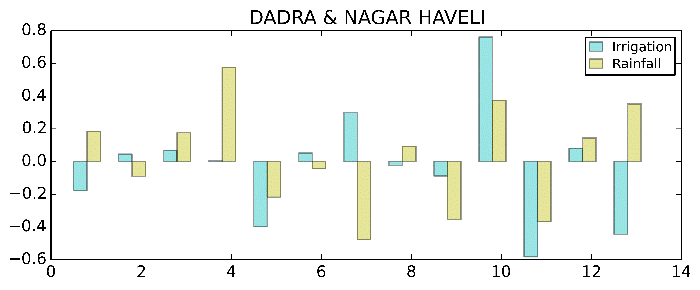

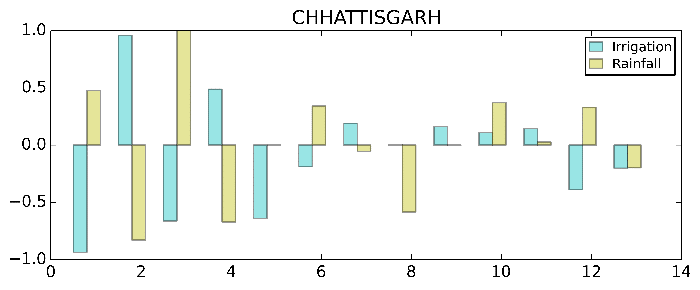

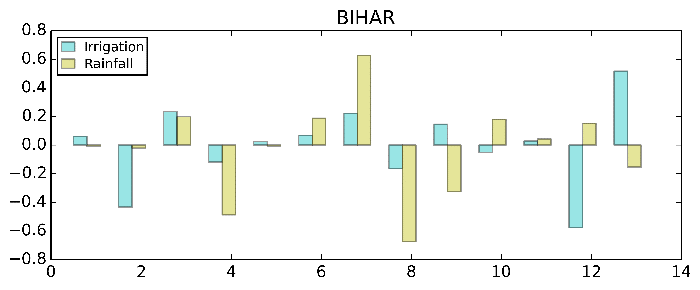

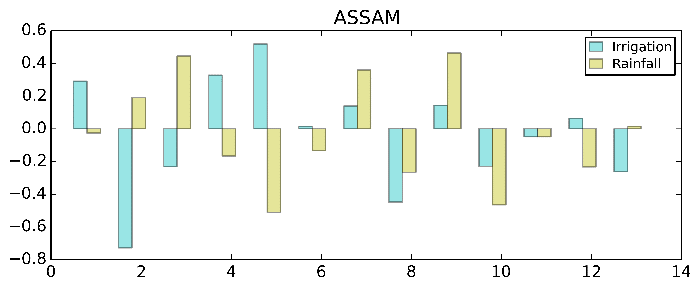

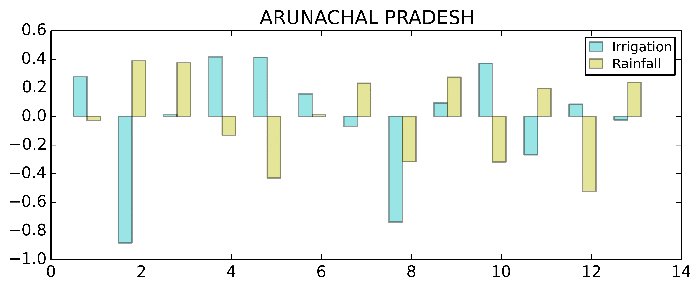

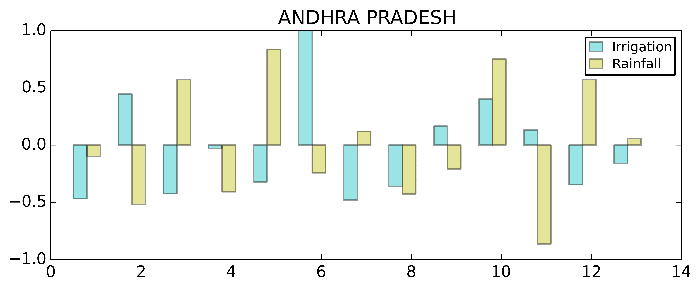


Figure S5: First difference of standardized anomaly of irrigated areas and rainfall for the selected states in India.
